# More Severe COVID-19 in Patients With Active Cancer: Results of a Multicenter Cohort Study

**DOI:** 10.3389/fonc.2021.662746

**Published:** 2021-05-07

**Authors:** Caterina Monari, Caterina Sagnelli, Paolo Maggi, Vincenzo Sangiovanni, Fabio Giuliano Numis, Ivan Gentile, Alfonso Masullo, Carolina Rescigno, Giosuele Calabria, Angelo Salomone Megna, Michele Gambardella, Elio Manzillo, Grazia Russo, Vincenzo Esposito, Clarissa Camaioni, Vincenzo Messina, Mariantonietta Pisaturo, Enrico Allegorico, Biagio Pinchera, Raffaella Pisapia, Mario Catalano, Angela Salzillo, Giovanni Porta, Giuseppe Signoriello, Nicola Coppola

**Affiliations:** ^1^ Infectious Diseases Unit, Department of Mental Health and Public Medicine, University of Campania “L. Vanvitelli”, Naples, Italy; ^2^ Infectious Diseases Unit, A.O. S Anna e S Sebastiano Caserta, Caserta, Italy; ^3^ Third Infectious Diseases Unit, AORN dei Colli, P.O. Cotugno, Naples, Italy; ^4^ Emergency Unit, PO Santa Maria delle Grazie, Pozzuoli, Italy; ^5^ Infectious Diseases Unit, University Federico II, Naples, Italy; ^6^ Infectious Diseases Unit, A.O. San Giovanni di Dio e Ruggi D’Aragona Salerno, Salerno, Italy; ^7^ First Infectious Diseases Unit, AORN dei Colli, PO Cotugno, Naples, Italy; ^8^ IX Infectious Diseases Unit, AORN dei Colli, PO Cotugno, Naples, Italy; ^9^ Infectious Disease Unit, A.O. San Pio, PO Rummo, Benevento, Italy; ^10^ Infectious Disease Unit, PO S. Luca, Vallo della Lucania, ASL Salerno, Vallo della Lucania, Italy; ^11^ IV Infectious Diseases Unit, AORN dei Colli, PO Cotugno, Naples, Italy; ^12^ Infectious Diseases Unit, Ospedale Maria S.S. Addolorata di Eboli, ASL Salerno, Eboli, Italy; ^13^ VIII Infectious Diseases Unit, AORN dei Colli, PO Cotugno, Naples, Italy; ^14^ Statistical Unit, Department of Mental Health and Public Medicine, University of Campania, Naples, Italy

**Keywords:** SARS-CoV-2, COVID-19, oncologic patients, severity disease, active cancer

## Abstract

**Background:**

The aim of the study was to compare coronavirus disease 2019 (COVID-19) severity presentation between oncologic and non-oncologic patients and to evaluate the impact of cancer type and stage on COVID-19 course.

**Methods:**

We performed a multicentre, retrospective study involving 13 COVID-19 Units in Campania region from February to May 2020. We defined as severe COVID-19 presentation the cases that required mechanical ventilation and/or admission to Intensive Care Units (ICU) and/or in case of death.

**Results:**

We enrolled 371 COVID-19 patients, of whom 34 (9.2%) had a history or a diagnosis of cancer (24 solid, 6 onco-hematological). Oncologic patients were older (p<0.001), had more comorbidities (p<0.001) and showed a higher rate of severe COVID-19 presentation (p=0.001) and of death (p<0.001). Compared to 12 patients with non-active cancer and to 337 without cancer, the 17 patients with active cancer had more comorbidities and showed a higher rate of severe COVID-19 and of mortality (all p values <0.001). Compared to the 281 non-severe patients, the 90 subjects with a severe presentation of COVID-19 were older (p<0.01), with more comorbidities (p<0.001) and with a higher rate of cancer (p=0.001). At multivariate analysis, age (OR 1.08, 95% CI: 1.04-1.11) and suffering from cancer in an active stage (OR 5.33, 95% CI: 1.77-16.53) were independently associated with severe COVID-19.

**Conclusions:**

Since the higher risk of severe evolution of COVID-19, cancer patients, especially those with an active malignancy, should be candidates for early evaluation of symptoms and early treatment for COVID-19.

## Background

The novel Coronavirus disease 2019 (COVID-19) caused by the Severe Acute Respiratory Syndrome CoronaVirus-2 (SARS-CoV-2) started in Wuhan, China, in December 2019 and has rapidly spread to a pandemic proportion worldwide (1–3). By the 9^th^ December 2020, 67,530,912 confirmed cases of COVID-19 and 1,545,140 related deaths have been reported to the World Health Association (WHO) (4).

SARS-CoV-2 is a zoonotic beta-coronavirus transmitted from human-to-human through nasal or oral droplets or through close contacts; fecal-oral transmission has a modest epidemiologic impact (5). After a mean incubation period of 5.2 (range 2-14) days (6), SARS-CoV-2 may lead to asymptomatic/mild forms in nearly 80% of infected subjects, to moderate forms in about 15% and to a severe condition in the remaining 5% of subjects (7). The severe forms are characterized by interstitial pneumonia frequently evolving into acute respiratory distress syndrome (ARDS) with a mortality rate of 10% (5).

The elderly and patients with comorbidities (cardiovascular disease, arterial hypertension, diabetes mellitus, chronic lung disease, renal failure, cerebrovascular disease or malignancy) have shown more frequently serious complications requiring the admission to an Intensive Care Unit (ICU) (8–13).

Few data have been published on the impact of SARS-CoV-2 infection in patients with malignancies (5). However, although results are controversial, a worse outcome has been described among oncologic patients (5, 14, 15). Instead, data regarding whether the stage and type of malignancy may influence the outcome of COVID-19 are still few.

The aim of the study was to investigate the main characteristics of COVID-19 in terms of evolution and prognosis in a cohort of oncologic patients compared to non-oncologic and to evaluate the impact of the type (solid *versus* onco-hematological cancer) and stage (active *vs* non active disease) in a regional cohort of patients with SARS-CoV-2 infection.

## Methods

### Study Design and Setting

We performed a multicentre, retrospective cohort study involving 13 COVID-19 Units in seven cities in the Campania region in southern Italy: Naples, Caserta, Salerno, Benevento, Pozzuoli, Eboli and Vallo della Lucania.

The study population included all adult patients (≥18 years) with a diagnosis of SARS-CoV-2 infection confirmed by a positive real time-polymerase chain reaction (RT-PCR) on naso-oropharyngeal swab, symptomatic or asymptomatic, evaluated at one of the centres participating in the study. The study period was from February 28^th^ to May 31^st^ 2020. We included both the patients admitted to hospital or receiving care at home. Exclusion criteria included minority age, not availability of clinical data and the absence of informed consent.

No study protocol or guidelines regarding the criteria of hospitalization and treatment recommendations were shared among the centres involved in this study. Patients were hospitalized and antiviral treatments were started according to the decision of physicians of each centre.

The study was approved by the Ethics Committee of the University of Campania L. Vanvitelli, Naples (n°10877/2020). All procedures performed in this study were in accordance with the ethics standards of the institutional and/or national research committee and with the 1964 Helsinki declaration and its later amendments or comparable ethics standards.

### Data Collection

All demographic, clinical, laboratory and radiological data and therapy details of both the hospitalized and non-hospitalized patients were collected in a database that we created at the end of February 2020 when the infection by SARS-CoV-2 started to spread in our area. From this database we extrapolated data regarding patients with a new diagnosis or a history of solid cancer or hematological malignancies and those without.

### Definitions and Sample Size

Microbiological diagnosis of SARS-CoV-2 infection was defined as a positive RT-PCR test on naso-oropharyngeal swab.

We defined patients with mild, moderate, or severe disease according to the clinical presentation of COVID-19. Precisely, patients with a mild infection were asymptomatic or experienced a mild infection with home quarantine and/or did not need oxygen (O_2_) therapy and/or had a Modified Early Warning Score (MEWS) below 3 points. Patients with a moderate infection were hospitalized and required low flow O_2_ therapy or non-invasive O_2_ therapy and/or had a MEWS equal or above 3 points (≥3). Lastly, patients with a severe infection needed management in an ICU and/or mechanical ventilation; in this definition we also included patients who died.

We defined patients with active cancer the subjects who had received anti-neoplastic treatment (radio-, chemo- or immuno/target-therapy or surgery) within the last 30 days or the subjects without indication of treatment due to a late stage of disease or without treatment options (off-therapy).

We defined patients with inactive cancer the subjects with a history of cancer without anticancer treatment in the last 30 days (follow-up).

Given the previously reported severity rate of COVID-19 of 33% in cancer patients and 10% in non-cancer patients in real-life settings (16), we calculated that a sample size of 30 subjects in a cancer-group and 301 in a non-cancer-group achieves 80% power to detect a difference between the groups of 23%. The statistic test used is the two-sided Fisher’s Exact test.

### Statistical Analysis

For the descriptive analysis, categorical variables were presented as absolute numbers and their relative frequencies. Continuous variables were summarized as mean and standard deviation if normally distributed or as the median and interquartile range (IQR) if not normally distributed. We performed a comparison of patients with active cancer, inactive cancer and without cancer using ANOVA for normally distributed variables and the Kruskal-Wallis test for non-normally distributed variables. Moreover, to compare solid and hematological cancer patients we performed Fisher-Freeman Halton exact test for categorical variables and Student’s t- or Mann-Whitney tests for continuous variables.

Odds ratios with 95% confidence intervals (CI) were estimated by a multiple logistic regression to identify independent factors related to clinically severe (management in ICU and/or need for mechanical ventilation and/or death) and non-severe (mild or moderate) clinical presentation of COVID-19. COVID-19 clinical presentation was the dependent variable, whereas age, sex, Comorbidity Index Score, and cancer stage were the covariates, since they proved significant at the univariate analysis.

To analyze the impact of co-morbidities, in the multivariate analysis we used the Charlson Index Score as a categorical variable (< 2 or ≥ 2 points), since the median Charlson Index Score in the population enrolled was 2 points.

A p-value below 0.05 was considered statistically significant. Analyses were performed using SPSS 23.0 (IBM, Armonk, NY, USA).

## Results

**Figure 1:**
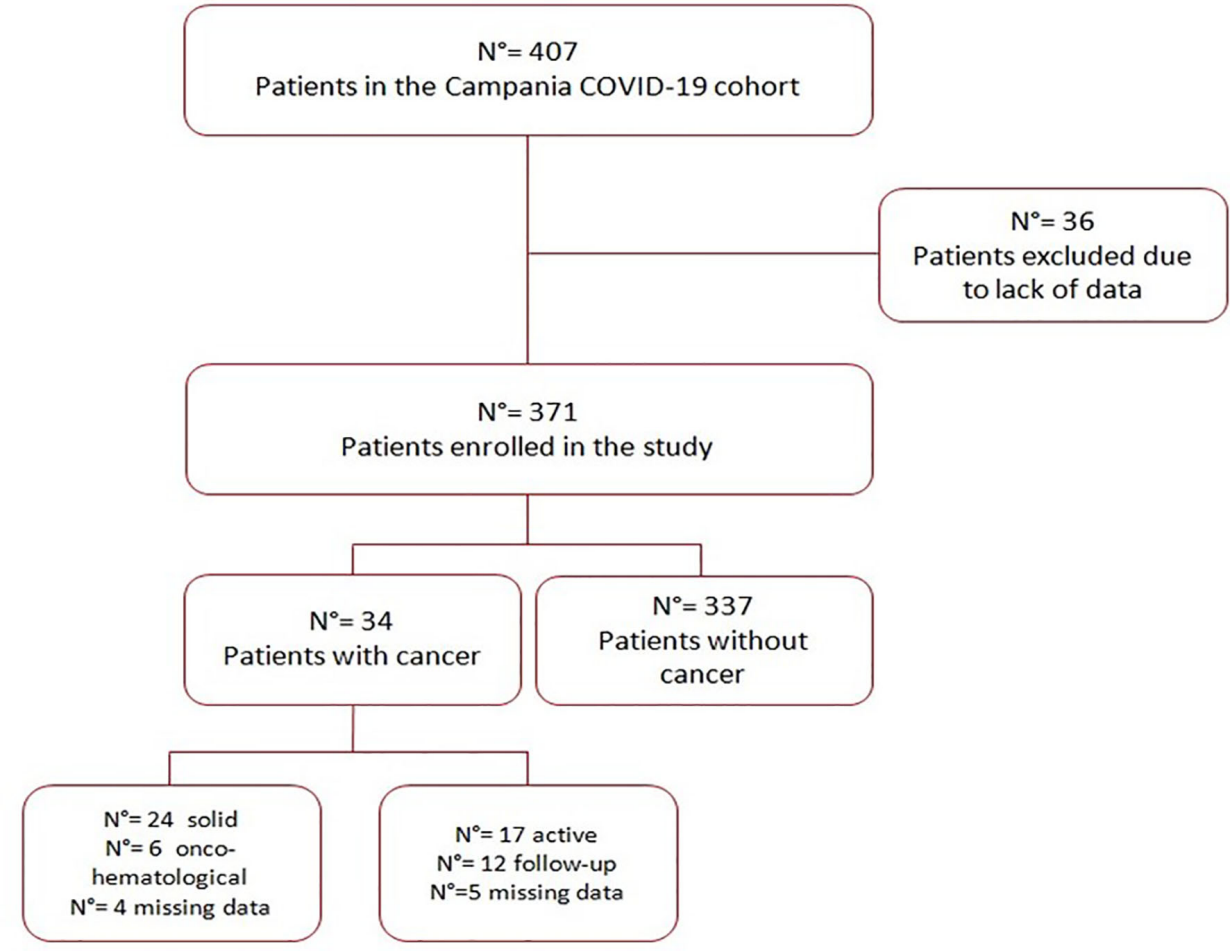
Study population flow-chart.

Of a total of 407 patients enrolled in the Campania COVID-19 cohort, only 371 reported data regarding the presence or absence of cancer and were enrolled in the present study, whereas 36 patients were excluded because of missing information. The study population flow-chart is shown in **Figure 1**.

We did not observe any difference in terms of demographic and clinical characteristics between patients included in the study and those excluded (data not shown).

Of the 371 patients enrolled, 34 (9.2%) had a history or a new diagnosis of cancer and 337 did not. Demographic and clinical characteristics of the 34 patients with cancer are shown in **Table 1**.

**Table 1:** The demographic and clinical characteristics of the 34 patients with cancer.

DEMOGRAPHIC VARIABLES	
Males, N° (%)	25 (73.5)
Age, years; median (IQR)	72 (63.5-78)
Days of enrolment after onset of symptoms; median (IQR) ^a^	4 (2-8)
N° (%) of subjects with nosocomial acquisition	2 (5.9)
N° (%) of subjects with contacts with suspected or confirmed COVID-19 cases	13 (38.2)
**CLINICAL VARIABLES**	
Charlson co-morbidity index, median (IQR)	6 (5-7.8)
N (%) of subjects with underlying chronic disease:	
- with hypertension - with cardio-vascular disease - with diabetes mellitus - with chronic kidney disease (CKD) - with chronic obstructive pulmonary disease (COPD) - with liver cirrhosis	19 (55.9) 14 (41.2) 9 (26.5) 8 (23.5) 6 (17.6) 2 (5.9)
**MALIGNANCY VARIABLES**	
Type of cancer, N° (%)	
- Prostatic cancer - Colon cancer - Breast cancer - Lung cancer - Gastric cancer - Pancreatic cance - Melanoma - Liver cancer - Lymphoma - Myeloma - Leukemia - Womb cancer - Other - Not available	6 (17.6) 5 (14.7) 1 (2.9) 4 (11.8) 1 (2.9) 2 (5.9) 1 (2.9) 1 (2.9) 3 (8.8) 1 (2.9) 2 (5.9) 1 (2.9) 2 (5.9) 4 (11.8)
State of cancer disease, N° (%) ^b^:	
- in treatment - off-therapy - in post-treatment follow-up	12 (35.3) 5 (14.7) 12 (35.3)
History of previous cancer treatment, N° (%):	
- Surgery - Surgery in the previous 30 days - Chemotherapy - Chemotherapy in the last 30 days - Radiotherapy - Radiotherapy in the last 30 days - Immuno/Target-therapy - Immuno/Target-therapy in the last 30 days	14 (41.2) 5 11 (32.6) 4 5 (14.7) 1 2 (5.9) 2

a: not available data for 9 subjects; b: not available data for 5 subjects.

Oncologic patients were predominantly males (73.5%) and elderly (median age 72 years, interquartile range, IQR, 63.5-78). Several patients suffered from comorbidities, according to the Charlson Index Score, in particular arterial hypertension (55.9%) and cardio-vascular diseases (41.2%). The main cancer reported was prostatic cancer (17.6% of cases). Moreover, half of the patients (17/34) had active cancer (12 in chemo- or radio- or immuno-therapy and 5 in off-therapy), whereas 12 patients were in follow-up. Data regarding cancer stage was missing in 5 patients. The characteristics of patients with or without cancer are shown in **Supplementary Table 1**.

Among the oncologic patients, 24 subjects had solid and 6 subjects onco-hematological cancer. No significant difference between the two groups of patients were observed (**Supplementary Table 2**). The data on the phase of cancer disease were available for 29 patients: 17 patients had active cancer and 12 inactive. **Table 2** shows demographic and clinical data in the 17 subjects with active cancer, in the 12 with inactive cancer and in the 337 without cancer. Patients with active cancer had more comorbidities according to the Charlson index score [median score 7 (6-10) *vs* 5.5 (4-6) *vs* 2 (0-3) points respectively, p<0.001], in particular cardio-vascular disease (p<0.001). Moreover, they showed a higher rate of severe COVID-19 presentation (64.7% *vs* 16.7% vs 21.7%, p<0.001) and of mortality (52.9% *vs* 16.7% *vs* 13.4%, p<0.001) (**Table 2**).

**Table 2:** Comparison of patients with active cancer vs patients with cancer in the follow-up vs patients without cancer.

	Active cancer	Cancer in follow-up	Non-cancer	p-value
N° of subjects (%)	17	12	337	
Age, years; median (IQR)	72.0 (61-78)	71.5 (67-74.5)	58.0 (46-69)	<0.001
N° (%) of males	13 (76.5)	7 (58.3)	208 (61.7)	0.45
Charlson comorbidity index, median (IQR)	7.0 (6-10)	5.5 (4-6)	2 (0-3)	<0.001
N° of subjects with Charlson Index Score:				<0.001
- 0-1 - 2-3 - ≥ 4	0 (0.0) 2 (11.8) 15 (88.2)	0 (0.0) 1 (8.3) 11 (91.7)	161 (47.8) 103 (30.6) 73 (21.7)	
N° (%) of subjects with underlying chronic disease:				
- Arterial hypertension - Cardio-vascular disease - COPD	11 (64.7) 11 (64.7) 5 (29.4)	7 (58.3) 1 (8.3) 1 (8.3)	138 (40.9) 71 (21.1) 47 (13.9)	0.083 <0.001 0.17
N° (%) of patients with solid cancer	13 (76)	10 (83)	–	0.65
Symptoms, n (%):				
- Fever - Dyspnea - Anosmia - Ageusia - Cough - Diarrhea - Skin Lesions	6 (37.5) 6 (37.5) 1 (9.1) 1 (9.1) 6 (37.5) 0 (0.0) 0 (0.0)	7 (63.6) 5 (45.5) 0 (0.0) 1 (16.7) 6 (54.5) 2 (18.2) 0 (0.0)	206 (64.6) 142 (44.4) 42 (17.7) 50 (21.5) 142 (44.4) 24 (9.2) 3 (1.6)	0.023 0.86 0.40 0.59 0.68 0.29 0.89
N° (%) of subjects with ARDS	2 (14.3)	0 (0.0)	31 (12.7)	0.59
Clinical presentation, N° (%):				<0.001
- mild - moderate - severe	1 (5.9) 5 (29.4) 11 (64.7)	2 (16.7) 8 (66.7) 2 (16.7)	141 (41.8) 123 (36.5) 73 (21.7)	
N° (%) of patients who died	9 (52.9)	2 (16.7)	45 (13.4)	<0.001
N° (%) of patients receiving corticosteroids	4 (23.5)	4 (33.3)	31 (9.2)	0.006
N° (%) receiving O2 therapy	12 (70.6)	9 (75.0)	195 (57.9)	0.30

Of the total 371 subjects enrolled, 90 patients (24.3%) showed severe COVID-19 presentation and 281 did not. Demographic and clinical characteristics of the two groups of patients are shown in **Table 3**. The 90 patients with a severe presentation of COVID-19 were older [median (IQR) 71.5 (57-80) vs 57 (45-66) years, p<0.01], had more comorbidities [median score (IQR) 4 (2-6) *vs* 0 (1-3) points, p<0.001), in particular cardio-vascular disease, arterial hypertension and chronic kidney disease (CKD), and had a higher rate of cancer (18.9 *vs* 6.0%, p=0.001) (**Table 3**).

**Table 3:** Comparison of patients with severe and non-severe clinical presentation of COVID-19.

	Severe N= 90 (24.3%)	Non-severe N= 281 (75.7%)	P value
N° (%) of males	64 (71.1)	169 (60.1)	0.08
Age, years; median (IQR)	71.5 (57-80)	57 (45-66)	<0.001
Charlson co-morbidity index; median (IQR)	4 (2-6)	1 (0-3)	<0.001
N° (%) of subjects with Charlson index:			<0.001
- 0-1 - 2-3 - ≥ 4	20 (22.2) 22 (24.5) 48 (53.3)	141 (50.2) 84 (29.9) 56 (19.9)	
N (%) of subjects with underlying chronic disease:			
- arterial hypertension - cardio-vascular disease - diabetes - chronic kidney disease - COPD - liver cirrhosis	50 (55.6) 35 (38.9) 20 (22.2) 13 (14.4) 16 (17.8) 3 (3.3)	107 (38.1) 50 (17.8) 38 (13.5) 18 (6.4) 37 (13.2) 4 (1.4)	0.005 <0.001 0.065 0.026 0.3 0.4
N° of subjects: - with solid cancer - with onco-hematological cancer - not available data	17 (26.6) 12 2 3	17 (6.0)12 4 1	0.001 0.4 0.66 -
Cancer stage, N° (%):			
- Active - In follow-up	11 (12.2)10 (11.1)	6 (2.1)2 (0.7)	0.020.01
Symptoms, N° (%):			
- Fever - Dyspnea - Anosmia - Ageusia - Cough - Diarrhea - Skin lesions	62 (68.8) 54 (60) 2 (2.2) 6 (6.7) 46 (51.1) 9 (10) 0	186 (66.2) 102 (36.3) 41 (14.6) 46 (16.4) 110 (39.1) 17 (6) 3 (1.1)	0.63 <0.001 0.014 0.23 0.02 0.07 1.0
N° (%) of subjects with ARDS	16 (17.8)	17 (6)	<0.001
N° (%) of hospitalized patients	90 (100)	227 (80.8)	<0.001
N° (%) of patients who died	52 (57.8)	7 (2.5)	<0.001
N° (%) of patients receiving corticosteroids	8 (8.9)	31 (11)	0.69
N° (%) receiving O2 therapy	85 (94.4)	135 (48)	<0.001

Lastly, the multivariate analysis demonstrated that factors independently associated with a severe form of COVID-19 were age (OR 1.08, 95% CI: 1.04-1.11, p<0.001) and suffering from cancer in an active stage (OR 5.33, 95% CI: 1.77-16.53, p<0.001) (**Table 4**).

**Table 4:** Multivariate analysis (logistic regression model) identifying factors independently associated with a severe presentation of COVID-19.

	OR (95% CI)	P value
**Sex (F vs M)**	0.7 (0.4-1.23)	0.22
**Age**	1.08 (1.04-1.11)	<0.001
**Charlson Index Score (≥ 2 vs <2 points)**	2.06 (0.84-5.04)	0.12
**Active cancer (*vs* inactive cancer and non-cancer)**	5.33 (1.72-16.53)	<0.001

## Discussion

Patients with cancer represent a highly vulnerable population. Our study showed that the presence of cancer in an active phase was independently associated with a poor prognosis of COVID-19. In fact, compared to the non-cancer patients and to those with previous cancer, patients with active cancer, defined as patients receiving anti-neoplastic treatment in the last 30 days or those without indication because of the late stage of the disease, showed a higher prevalence of severe COVID-19, expressed as ICU admission, invasive ventilation and/or death.

Several studies have described a more severe clinical presentation of COVID-19 in patients with cancer compared to those without (5, 14, 17–20), but the results are still controversial (21–25).

A recent meta-analysis enrolling 32 studies on 46,499 patients, of whom 1,776 with cancer, showed a higher rate of all-cause mortality (RR 1.66; 95% CI 1.33-2.07, p<0.0001), and a greater need for ICU admission (RR 1.56; 9%% CI 1.31-1.87, p<0.0001) in oncologic *versus* non-oncologic patients. However, a subgroup analysis defined that among the patients above 65 years, all-cause mortality was similar between subjects with or without cancer (23). These interesting data may be explained by the fact that increased age was a risk factor for a poor outcome itself (15) among patients with COVID-19 or because older subjects are characterized by an increased prevalence of comorbidities (23, 26).

Few data with controversial results are available on whether the severity of COVID-19 may be influenced by the cancer stage, and different definitions of active cancer have been described (17, 20).

A multicenter cohort study on 928 patients with active or previous cancer with confirmed SARS-CoV-2 infection (CCC19, COVID-19 and Cancer Consortium database) reported a 30-day all-cause mortality of 13% and, among the factors independently associated with mortality, the presence of an active cancer (OR 5.20, 995% CI 2.77-9.77) (20).

A retrospective case study by Zhang et al. reported a mortality rate of 28.6% in 28 cancer patients with COVID-19 and confirmed a higher risk of developing severe events in those patients who received antitumor treatments within 14 days from the diagnosis (hazard ratio, HR=4.079, 95% CI 1.086-15.322, p=0.037) (17).

On the contrary, a prospective observational study performed in a cohort of 800 patients with a diagnosis of cancer and COVID-19 in the UK did not find any significant effect of chemotherapy (received in the past 4 weeks) on the mortality rate from COVID-19, even after adjusting the analysis for age, gender and comorbidities (OR 1.18; 95% CI 0.81-1.72, p=0.38) (21).

Also, a recent cohort study performed in 2 hospitals in New York found no significant differences in mortality between patients with active cancer and non-cancer patients (p=0.894), suggesting that a diagnosis of active cancer alone and recent anti-tumor treatment do not predict a worse COVID-19 outcome (22).

Some authors suggested a higher prevalence of severe forms of COVID-19 in patients with hematological cancers compared to those with solid cancers (6). Meng et al. reported a worse clinical outcome with twice the mortality rate (50 *vs* 26.1%) in subjects with hematological *vs* solid malignancy, although not statistically significant (p=0.06), but no differences in terms of severity (19).

This study has some limitations: the retrospective design, the small sample size of cancer patients and potential information biases. The strength of the study is its population representativeness in a low-intermediate endemic area of southern Italy, since 13 regional COVID-Units, both first and second level units, participated in the study.

In conclusion, the present study suggests that COVID-19 may have a more severe presentation in oncologic patients, in particular in those with active malignancy. These data may be explained by the fact that patients with active cancer, in off-therapy or in treatment, were vulnerable patients, with a higher risk of COVID-19 complications due both to the underlying disease and to the withdrawal of oncological therapy.

Despite some limitations, it represents an important preliminary contribution to our knowledge and understanding of COVID-19 in cancer patients. In fact, since the high risk of a severe evolution of COVID-19, especially in the active phase of the disease, cancer patients should receive special attention and should be candidates for an early evaluation of symptoms suspecting COVID-19 and for early treatment. These results may help Health Authorities in the establishment of pathways tailored for oncologic patients with COVID-19. Nevertheless, many questions regarding the clinical management of this vulnerable population remain unanswered, in particular whether and when to start, withdraw or postpone anti-neoplastic treatments. A multidisciplinary approach with the collaboration of different professionals (oncologists, hematologists, infectious disease specialists, pulmonologists) is important to improve the patient’s prognosis. Other larger studies and longer follow-up are needed to better describe the effect of COVID-19 on oncologic patients and to understand when to start or continue cancer specific therapies.

## Data Availability Statement

The raw data supporting the conclusions of this article will be made available by the authors, without undue reservation.

## Ethics Statement

The study was approved by the Ethics Committee of the University of Campania L. Vanvitelli, Naples (n°10877/2020). All procedures performed in this study were in accordance with the ethics standards of the institutional and/or national research committee and with the 1964 Helsinki declaration and its later amendments or comparable ethics standards. Informed consent was obtained from all participants included in the study.

## Author Contributions

CM, CS, and NC were involved in study concept and design and drafting of the manuscript. PM, VS, FN, and IG were involved in critical revision of the manuscript for important intellectual content. AMa, CR, GC, AMe, MG, EM, GR, VE, CC, VM, MP, EA, BP, RP, MC, AS, and GP were involved in acquisition of data, analysis and interpretation of data and in critical revision of the manuscript. GS performed the statistical analysis. Campania COVID-19 group was involved in the enrolment of the patients. All authors contributed to the article and approved the submitted version.

## Funding

“POR Campania FESR 2014-2020-Avviso per l’acquisizione di manifestazioni di interesse per la realizzazione di servizi di ricerca e sviluppo per la lotta contro il Covid-19 (DGR n. 140 del 17 marzo 2020), Project: “IDENTIFICAZIONE DEI FATTORI DEMOGRAFICI, CLINICI, VIROLOGICI, GENETICI, IMMUNOLOGICI E SIEROLOGICI ASSOCIATI AD OUTCOME SFAVOREVOLE NEI SOGGETTI CON COVID-19”, Regione Campania, Italy.

## Conflict of Interest

The authors declare that the research was conducted in the absence of any commercial or financial relationships that could be construed as a potential conflict of interest.

## Abbreviations

COVID-19, Coronavirus disease 2019; SARS-CoV-2, Severe Acute Respiratory Syndrome CoronaVirus-2; WHO, World Health Association; ICU, intensive care unit; MEWS, Modified Early Warning Score; IQR, interquartile range; OR, Odds ratios; IC, confidence intervals.
