# Supplementary material for: More Severe COVID-19 in Patients With Active Cancer: Results of a Multicenter Cohort Study
Source: Front Oncol. 2021 May 7;11:662746. doi: 10.3389/fonc.2021.662746 (PMC8139554; doi:10.3389/fonc.2021.662746)
Supplement: Supplementary file 1 [file Table_1.docx]

**Supplementary Table 1**: Demographic and clinical characteristics of the patients according to the presence or absence of malignancy

|  | **With cancer**  **N=34 (9.2%)** | **Without cancer**  **N=337 (90.8%)** | **P**  **value** |
| --- | --- | --- | --- |
|  |  |  |  |
| Males, N° (%) | 25 (73.5) | 208 (61.7) | **0.197** |
| Age, years, median (IQR) | 72 (63.5-78) | 58 (46-69) | **<0.001** |
| Days of enrolment after onset of symptoms, median (IQR) | 5 (2-10) | 5 (3-8) | **0.4** |
| Charlson co-morbidity index, median (IQR) | 6 (5-7.8) | 2 (0-3) | **<0.001** |
| N° (%) of subjects with Charlson index:   - 0-1 - 2-3 - ≥ 4 | 0 (0)  3 (8.8)  31 (91.2) | 161 (47.8)  103 (30.6)  73 (21.6) | **<0.001** |
| N (%) of subjects with underlying chronic disease:   - with hypertension   - with cardio-vascular disease   - with diabetes - with chronic kidney disease - with COPD - with liver cirrhosis | 19 (55.9)  14 (41.2)  9 (26.5)  8 (23.5)  6 (17.6)  2 (5.9) | 138 (40.9)  71 (21.1)  49 (14.5)  23 (6.8)  47 (13.9)  5 (1.5) | **0.1**  **0.01**  **0.08**  **0.004**  **0.6**  **0.13** |
| N° (%) of symptomatic subjects | 34 (100%) | 327 (97%) | **0.3** |
| N° (%) of subjects with:   - fever - cough - dyspnea   - ageusia   - anosmia - diarrhea - cutaneous lesions | 18 (52.9)  14 (41.2)  14 (41.2)  2 (5.9)  1 (2.9)  2 (5.9)  0 (0) | 230 (68.2)  142 (42.1)  142 (42.1)  50 (14.8)  42 (12,5)  24(7.1)  3 (12.5) | **0.1**  **1.0**  **1.0**  **0.53**  **0.32**  **1.0**  **1.0** |
| N° (%) of subjects with ARDS | 2 (5.9) | 31 (9.2) | **1.0** |
| N° (%) of subjects treated with HCQ | 19 (55.9) | 187 (55.4) | **0.9** |
| N° (%) of subjects treated with PI | 13 (38.2) | 151 (40) | **0.46** |
| N° (%) of subjects treated with macrolide | 17 (50) | 140 (41.5) | **0.56** |
| N° (%) of subjects treated with tocilizumab or other biological drug | 2 (8) | 20 (5.9) | **0.99** |
| Imaging showing pneumonia | 13 (38) ^a^ | 111 (32.9) | **0.53** |
| N° (%) of hospitalized patients | 34 (100) | 283 (84) | **0.01** |
| N° (%) of pts with mild clinical presentation ^b^ | 4 (11.8) | 139 (41.2) | **0.001** |
| N° (%) of pts with moderate clinical presentation^c^ | 13 (38.2) | 124 (36.8) | **0.85** |
| N° (%) of pts with severe clinical presentation ^d^ | 17 (50) | 73 (21.7) | **0.001** |
| N° (%) of patients who died ^e^ | 14 (41.2) | 45 (13.4) | **<0.001** |
| N° (%) of patients receiving corticosteroids | 8 (23.5) | 31 (9.2) | **0.017** |
| N° (%) of subjects receiving O_2_ therapy | 25 (73.5) | 195 (58.4) | **0.09** |

COPD: chronic obstructive pulmonary disease; ARDS: acute respiratory distress syndrome; HCQ: hydroxychloroquine; PI: protease inhibitor

a: data not available for 16 patients

b: at home isolation and/or not-O2 therapy and/or MEWS<3

c: non-ventilation- O2 therapy and/or MEWS>3

d: mechanical ventilation and/or management in ICU and/or death

e: data not available for 7 oncologic and 24 non-oncologic patients

**Supplementary Table 2**: Demographic and clinical characteristics of patients according to the type of cancer (solid or onco-hematological)

|  | **Solid cancer** | **Onco-hematological cancer** | **p value** |
| --- | --- | --- | --- |
| N° of subjects | 24 | 6 |  |
| N° (%) of males | 17 (70.8) | 4 (66.7) | **0.43** |
| Age, years; median (IQR) | 72 (65-75.8) | 65 (56.5-72.8) | **0.18** |
| Days of enrolment after onset of symptoms; median (IQR) | 3.5 (0.5-6.5) | 9 (5-13) | **0.19** |
| Charlson co-morbidity index, median (IQR) | 6 (5,75;8) | 6 (5;8) | **0.13** |
| N° (%) of subjects with Charlson Index Score:   - 0-1 - 2-3 - ≥ 4 | 0 (0)  1 (4.2)  23 (95.8) | 0 (0)  2 (33.3)  4 (66.7) | **0.06** |
| N (%) of subjects with underlying chronic disease:   - Arterial hypertension - Cardio-vascular disease - Diabetes mellitus - Chronic Kidney Disease - COPD - Liver cirrhosis | 16(66.7)  11(45.8)  9 (37.5)  8 (33.3)  6 (25.0)  2 (8.3) | 2 (33.3)  2 (33.3)  0 (0)  0 (0)  0 (0)  0 (0) | **0.14**  **0.67**  **0.078**  **0.1**  **0.2**  **0.6** |
| **N° (%) of symptomatic subjects** | 24 (100) | 6 (100) |  |
| N° (%) of subjects with:   - Fever - Cough - Dyspnea - ageusia   - anosmia   - Diarrhea - Skin lesions | 10 (41.7)  8 (33.3)  7 (29.2)  2 (8.3)  1 (4.2)  2 (8.3)  0 (0) | 5 (83.3)  4 (66.7)  5 (83.3)  0 (0)  0 (0)  0 (0)  0 (0) | **0.07**  **0.3**  **0.059**  **1.0**  **1.0**  **1.0**  **/** |
| Treatments regimen, N° (%):   - HCQ - PI - Macrolide - Tocilizumab or other biological drug | 14 (58.3)  10 (41.7)  13 (54.2)  1 (4.2) | 4 (66.7)  3 (50)  2 (33.3)  1 (16.7) | **0.11**  **0.71**  **0.36**  **0.27** |
| Imaging showing pneumonia, N° (%) | 11 (45.8) | 2 (33.3) | **0.58** |
| N° (%) of hospitalized patients | 24 (100) | 6 (100) | **1** |
| Days of hospitalization^a^, mean + SD | 22.3 ± 14.5 | 13 ± 6.2 | **0.069** |
| Clinical presentation, N° (%): |  |  |  |
| - Mild ^b^ - Moderate ^c^ - Severe ^d^ | 3 (12.5)  9 (37.5)  12 (50.0) | 0 (0)  4 (66.7)  2 (33.3) | **0.4**  **0.1**  **0.44** |
| N° (%) of subjects with active cancer ^e^ | 13 (54.2) | 4 (66.7) | **1.0** |
| Cancer stage, N° (%) ^f^:   - in therapy - off-therapy - in follow-up | 9 (37.5)  4 (16.7)  10 (41.7) | 3 (50)  1 (16.7)  2 (33.3) | **0.9** |
| N° (%) of patients who died ^e^ | 11 (45.8) | 1 (16.7) | **0.4** |
| N° (%) of patients receiving corticosteroids | 6 (25.0) | 2 (33.3) | **0.45** |
| N° (%) receiving O_2_ therapy | 17 (70.8) | 5 (83.3) | **0.8** |

COPD: chronic obstructive pulmonary disease; HCQ: hydroxychloroquine; PI: protease inhibitor

a: until discharge or death

b: at home isolation and/or not-02 therapy and/or MEWS<3

c: non-ventilation- 02 therapy and/or MEWS>3

d: ventilation 02 therapy and/or management in critical care unit and/or death

e: data not available for 6 subjects (5 with solid and 1 with onco-hematological cancer);

f: data not available for 1 subjects with solid cancer
